# Should I stay or should I go? Fitness costs and benefits of prolonged parent–offspring and sibling–sibling associations in an Arctic-nesting goose population

**DOI:** 10.1007/s00442-016-3595-4

**Published:** 2016-03-19

**Authors:** Mitch D. Weegman, Stuart Bearhop, Geoff M. Hilton, Alyn J. Walsh, Kaitlin M. Weegman, David J. Hodgson, Anthony David Fox

**Affiliations:** Centre for Ecology and Conservation, College of Life and Environmental Sciences, University of Exeter, Cornwall Campus, Penryn, TR10 9EZ UK; Wildfowl & Wetlands Trust, Slimbridge, Gloucester, GL2 7BT UK; National Parks and Wildlife Service, Wexford Wildfowl Reserve, North Slob, Wexford, Ireland; Department of Biosciences, University of Exeter, Streatham Campus, Exeter, EX4 4QD UK; Department of Bioscience, Aarhus University, Kalø, Grenåvej 14, 8410 Rønde, Denmark

**Keywords:** Bayesian multistate model, Cost–benefit model, Fitness, Greenland white-fronted goose, Breeding probability, Long-term family relationship

## Abstract

Theory predicts persistence of long-term family relationships in vertebrates will occur until perceived fitness costs exceed benefits to either parents or offspring. We examined whether increased breeding probability and survival were associated with prolonged parent–offspring and sibling–sibling relationships in a long-lived Arctic migrant herbivore, the Greenland white-fronted goose (*Anser albifrons flavirostris*). Although offspring associated with parents for 1–13 years, 79 % of these associations lasted two or less years. Only 65 (9.9 %) of the 656 marked offspring bred once in their lifetime, and just 16 (2.4 %) bred twice or more. The probability of birds with siblings breeding successfully in a subsequent year was credibly greater than that of independent birds at ages 5, 6, and 7. Survival of offspring with parents was credibly greater than that of independent/nonbreeder birds at all possible ages (i.e., ages 2–7+). A cost–benefit matrix model utilizing breeding and survival probabilities showed that staying with family groups was favored over leaving until age 3, after which there were no credible differences between staying and leaving strategies until the oldest ages, when leaving family groups was favored. Thus, most birds in this study either departed family groups early (e.g., at age 2, when the “stay” strategy was favored) or as predicted by our cost–benefit model (i.e., at age 3). Although extended family associations are a feature of this population, we contend that the survival benefits are not sufficient enough to yield clear fitness benefits, and associations only persist because parents and offspring mutually benefit from their persistence.

**Electronic supplementary material:**

The online version of this article (doi:10.1007/s00442-016-3595-4) contains supplementary material, which is available to authorized users.

## Introduction

Prolonged associations among kin (particularly between parents and offspring) are likely to be maintained only as long as fitness costs and benefits favor all parties. Generally, increased parental investment improves offspring fitness (Trivers 1972; Cam et al. 2003; Tinkler et al. 2007) because prolonged parent–offspring associations contribute to offspring learning foraging strategies, predator awareness, migratory routes, and potentially reproductive tactics from parents (Hochbaum 1955; Raveling 1970; Owen 1980; Warren et al. 1993; Slagsvold and Wiebe 2011). Parents may benefit from offspring associations through enhanced reproductive success (e.g., “helpers;” Skutch 1961), while extended parent–offspring bonds contribute to mutual predator defense and/or greater foraging success, for example amongst African elephants (*Loxodonta africana*; Moss and Poole 1983), killer whales (*Orcinus orca*; Baird 2000), and sperm whales (*Physeter macrocephalus*; Whitehead et al. 1991). Other family associations, such as sibling–sibling associations, are less studied, but are thought to persist in geese because extended family associations increase group size, enhancing predator detection, social dominance, and access to resources (Boyd 1953; Raveling 1970; Black and Owen 1989a; Ely 1993; Warren et al. 1993; Fox et al. 1995).

However, benefits from prolonged associations diminish if they are maintained at increased cost to the individual’s own future reproductive success and survival (Stearns 1992). For offspring, remaining with parents into adulthood postpones breeding and hence investment in their own fitness. Theory therefore predicts that parent–offspring relationships are maintained until a net cost occurs either to parents, offspring, or both, at which point the association is terminated by one of the parties (Trivers 1974). Black and Owen (1989b) found no obvious fitness cost to extended parent–offspring associations in barnacle geese (*Branta leucopsis*), and proposed that adult offspring assist their parents for inclusive fitness benefits (i.e., increased fitness of group members as a result of individuals “helping” their parents; Hamilton 1964). Remaining with siblings after the termination of parental bonds may therefore also be favored because of the benefits of increased group size to inclusive fitness when siblings reproduce in subsequent years.

Offspring must determine the optimal duration of their association with parents, balancing the risk of dying before independence with the incremental future fitness gain from spending an additional period with parents (e.g., enhanced breeding probability when subsequently independent). Previous studies have assessed short-term fitness costs of extended family associations (Inger et al. 2010). Here, we describe the lifetime fitness consequences of long-term parent–offspring associations in Greenland white-fronted geese (*Anser albifrons flavirostris*), long-lived Arctic-nesting birds characterized by uniquely prolonged (up to 13 years) but highly variable kinship bonds (Warren et al. 1993), based on known-age marked individuals followed over many years at wintering sites across Great Britain and Ireland. In this paper, we address three main questions:

1. Do offspring associating with family members enjoy increased age-specific breeding probability when independent in subsequent years?
2. Is there a survival cost to independence, i.e., do offspring associating with parents or siblings exhibit greater age-specific survival than those that are independent?
3. Finally, are offspring balancing relationship costs and benefits via optimal bond duration to maximize fitness?

## Materials and methods

### Study area

Wexford Slobs (52°22′N, 6°24′W) in southeast Ireland comprise intensively managed grassland and cropland that constitute the single most important wintering area for Greenland white-fronted geese, supporting over one-third of the global population (Warren et al. 1992; Fox et al. 1998). From 1983 to 2003, 656 first-winter Greenland white-fronted geese were caught at Wexford using traditional cannon-netting techniques. We truncated the data set after the 2003 cohort to ensure adequate capture histories (i.e., compiled up to 2009) for later cohorts. Caught birds were individually marked with a white plastic leg band and an orange neck collar (both inscribed with the identical alphanumeric code; see Warren et al. 1992) as well as a standard numbered metal ring. Collar code combinations were visible from up to 800 m using a 20–60× spotting scope. Individual geese were aged on capture by plumage characteristics (presence/absence of white frons on face and black belly bars; Cramp and Simmons 1977) and sexed by cloacal examination (Warren et al. 1992). AJW resighted geese weekly during winter at Wexford from 1983 to 2010, beginning when birds arrived in autumn.

### Parent–offspring and sibling–sibling observations

Parent–offspring and sibling–sibling associations were determined by repeated observations (≥2) of collared individuals together within and among winters. Temporary relationships are hard to identify as some birds were rarely seen; thus, single resightings of associations may be unreliable and were not used (Owen 1984). Associations of focal individuals with unmarked birds were not considered because unmarked individuals could not be consistently identified within or between years. Breeding occurs at a low density over large areas (>15,000 km^2^) of remote west Greenland, so little research has been conducted on the breeding biology of Greenland white-fronted geese. Our winter observations of parent–offspring and sibling–sibling associations are contingent on (1) juveniles surviving as goslings, fledging, and migrating to wintering areas, and (2) family members remaining together through summer and autumn. Further, not all individuals of a family unit were captured and marked; thus, our estimates of family association duration are likely to be conservative with respect to the “true” association duration. In families comprising more than two adults, parents were identified through repeated observations in association with marked first winter birds, and other marked adult family group members were assumed to be offspring from a previous year still associating with parents; this method for determining relatedness of closely associating individuals has recently been verified using molecular genetics in light-bellied brent geese (*Branta bernicla hrota*; see Harrison et al. 2010). When previously associated birds were not resighted together over the course of a subsequent winter, the relationship was considered terminated and a cause assigned (i.e., focal bird not resighted, associate not resighted, both not resighted, both resighted but no longer associating). Adult paired geese resighted repeatedly (≥2 times) with juveniles within a winter were considered breeding parent birds. Twelve birds that paired with other collared birds were subsequently resighted with juveniles; in these cases, one bird of each pair was randomly removed from analyses.

### Statistical analyses

All birds were resighted in their first winter with at least one parent; the sum of years that a goose marked in their first winter associated with both parents and only one parent defined the duration of that parent–offspring association. Importantly, parents associating with adult offspring may breed in subsequent years, but associating offspring do not. Thus, only parents and independent offspring (i.e., not those associating with family) may accrue a direct reproductive benefit (through increased breeding probability) from familial association. We assumed offspring did not breed whilst associating with siblings, as we have no observations of this occurring.

To determine age-specific survival and breeding transition probabilities of birds with parents, siblings (post-parents), and those considered independent/nonbreeders and independent/breeders, we developed Bayesian multistate capture–recapture models using WinBUGS (http://www.mrc-bsu.cam.ac.uk/bugs), version 1.4.3, adapting examples outlined in Kéry and Schaub (2012) and Weegman et al. (2015, 2016). All models were run through the R2WinBUGS package (Gelman et al. 2013) in Program R, version 2.14.2 (R Development Core Team 2012). We assigned capture histories according to states: “1”—seen, with parents, “2”—seen, with ≥1 sibling, “3”—seen, independent/non-breeder, and “4”—seen, independent/breeder; only one state was assigned per year. In rare cases where states varied within year (i.e., multiple observations of birds with parents, siblings, and independent during the same winter), we used the modal state. All birds were resighted with at least one parent in their first year; thus, birds could not begin the capture history independent. Accordingly, survival probabilities of birds with a sibling, those that were independent/nonbreeder, and those that were independent/breeder were calculated from age 2, and the probability of transitioning from state “with parents” to all other states was calculated from age 1. Birds did not transition to previous states (e.g., from “independent/nonbreeder” to “with parents”). We limited multistate capture–recapture models to seven age classes, where ages of 7+ were combined into a single class due to small sample sizes. Previous age-specific survival analyses have indicated a linear relationship between age and survival (Weegman 2014); thus, to increase the precision of our estimates in the multistate framework, we modeled age as a linear trend on survival. We have no evidence to suggest a similar relationship between ages and transitions, so we modeled transitions with full age specificity (i.e., ages 1–7+). Nonetheless, we formed an additional multistate model with age-constant transitions, the results of which are presented in Figs. S1 and S2 in the Electronic supplementary material (ESM). To estimate age- and state-specific survival and transition probabilities, we used normally distributed, noninformative priors with mean = 0 and variance = 0.001 with the multinomial logit link function for all but one transition parameter, constrained so that their sum was <1; the last transition was calculated as

$$\beta_{n} = 1 - \mathop \sum \limits_{i = 1}^{n - 1} \beta_{i},$$

where *β*_*n*_ denotes the back-transformation of the final transition parameter *n*, based on back-transformations of other transitions, *β*_*i*_. To estimate the state-specific resighting probabilities, we used uniformly distributed, noninformative priors with mean = 0 and variance = 1. Posterior summaries from three Markov chain Monte Carlo (MCMC) chains were based on 450,000 iterations after a burn-in of 90,000 and a thinning interval of 10. We confirmed chain convergence using the Gelman–Rubin statistic (see Gelman and Rubin 1992), and greater than 8000 samples were drawn from posterior distributions. Posterior means are presented with 95 % credible intervals (CRI). Additional specification and code for the multistate model may be found in the ESM.

The multistate model produced posterior distributions of age- and state-specific probabilities of survival (modeled with age as a linear trend), and age-specific probabilities of moving between states (with parents, with siblings, independent/nonbreeder, independent/breeder). For each iteration of the model (i.e., each set of parameters in the posterior distribution), we populated an age- and state-transition matrix, where transitions to breeding were used as breeding probabilities. After transitioning into the breeding population, birds were subsequently lost to population growth in order to reflect the extreme rarity of multiple breeding attempts (this paper; Weegman 2014). Our proxy for fitness of the “wild-type” life history was the dominant eigenvalue of this transition matrix (*λ*_wt_), which gained its own posterior distribution via calculation across all iterations of the MCMC model. Hence, all projection matrices presented here are simplified versions of reality, in which “breeding” simply contributes a fecundity of 1, whereby *λ*_wt_ (in this case) is a measure of relative fitness that assumes clutch size and fledging success are independent of parental age. We tested whether the distributions for age-specific survival of birds with parents or siblings and those independent were credibly different (i.e., whether the 95 % CRI of the difference overlapped zero). Likewise, we tested whether the transitions to breeding were credibly different between birds with siblings and those independent. We report approximate *P*-values for these tests, citing the proportion of posteriors lying below zero for independent birds. We claim “credibility” when this proportion is either <0.05 or >0.95.

Using age-specific breeding probabilities and survival estimates of birds with parents, siblings, and independents, we then formed a cost–benefit matrix model to examine optimal bond durations and determine how fitness depends on the tradeoff between survival benefits of staying with family, and the breeding benefits of independence. The structure of the model was based on the assumption that at each age, birds have the choice to remain another year with their parents or leave; once birds left their parents, they had a similar choice to remain with their siblings or leave. Thus, we simulated all combinations of potential family outcomes: birds could leave parents aged 1, 2, …, 7+ years, then bond with siblings for 1, …, 7+ years or enter the nonbreeding independent state. Independent birds then enjoyed the observed age-specific probability of entering the independent breeding state, at which point they contributed unit recruitment to the population. For each simulation, we forced all birds to transition into independence from parents, and independence from siblings, at a fixed age, but applied the observed probabilities of survival and breeding. Our proxy for fitness was the dominant eigenvalue (*λ*_s_) of the age- and state-transition matrix formed by the simulated probabilities of state transitions and the observed probabilities of age- and state-specific survival. We used the posterior distributions of age- and state-specific survival to yield posterior distributions of simulated fitness. To determine the fitness costs or benefits associated with each simulated strategy, we calculated posterior distributions of the difference in fitness between wild-type and simulated strategies (*ω* = *λ*_wt_ −* λ*_s_). We also tested whether the simulated and wild-type distributions were credibly different (i.e., whether the 95 % CRI of* ω* overlapped zero). Similar to tests among survival and breeding transitions, we report approximate *P*-values for these tests, citing the proportion of wild-type posteriors lying below zero (claiming “credibility” when this proportion is either <0.05 or >0.95). We monitored correlations among parameter estimates of subsequent ages for each state to ensure that negative correlations were not influencing year-on-year cost–benefit calculations. We predicted an “intersection” age where the advantage to the individual would switch between “stay” and “leave” strategies, whereby the stay strategy would be favored for a few years (i.e., simulated fitness distributions would be credibly less than wild-type fitness distributions) until declining survival and/or perceived fitness gains would favor adoption of the leave strategy (i.e., simulated and wild-type distributions would be similar).

## Results

**Fig. 1:**
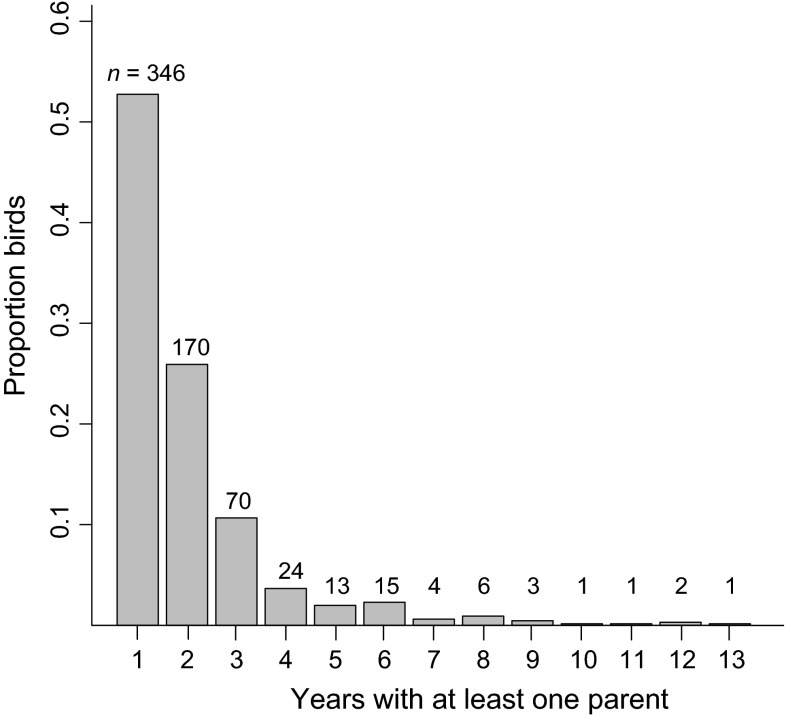
Durations (years) of parent–offspring relationships in Greenland white-fronted geese marked at Wexford, Ireland in 1983–2003 (*n* subset shown* above* *bars*)

For marked first-winter individuals that hatched from 1983 to 2003, duration of parent–offspring association varied from 1 to 13 years, although most (89 %) associations lasted 3 years or less (Fig. 1). The majority of the birds (78 %) did not associate with siblings upon becoming independent from their parents. Among those that associated with at least one sibling post-parents, durations varied from 1 to 13 years, although most (91 %) were 3 years or less (Fig. S3 in the ESM). Of 656 life histories of geese marked in their first winter, only 65 birds (9.9 %) bred successfully (i.e., were observed with young on wintering areas) at least once in their lives, 13 (1.9 %) bred successfully twice, and just three bred successfully three times (Weegman 2014). Among breeders and nonbreeders, observed mean duration of parent–offspring association was 2.31 (±SE 0.15) and 1.96 (±0.05) years, respectively. Mean observed duration of association with at least one sibling post-parents among breeders and non-breeders was 1.22 (±0.31) and 0.37 (±0.07) years, respectively. No geese were marked in 2000, and none bred from the 1996 (*n* = 22), 1997 (*n* = 13), 1999 (*n* = 13), and 2003 (*n* = 39) cohorts.

### State-specific demography

**Fig. 2:**
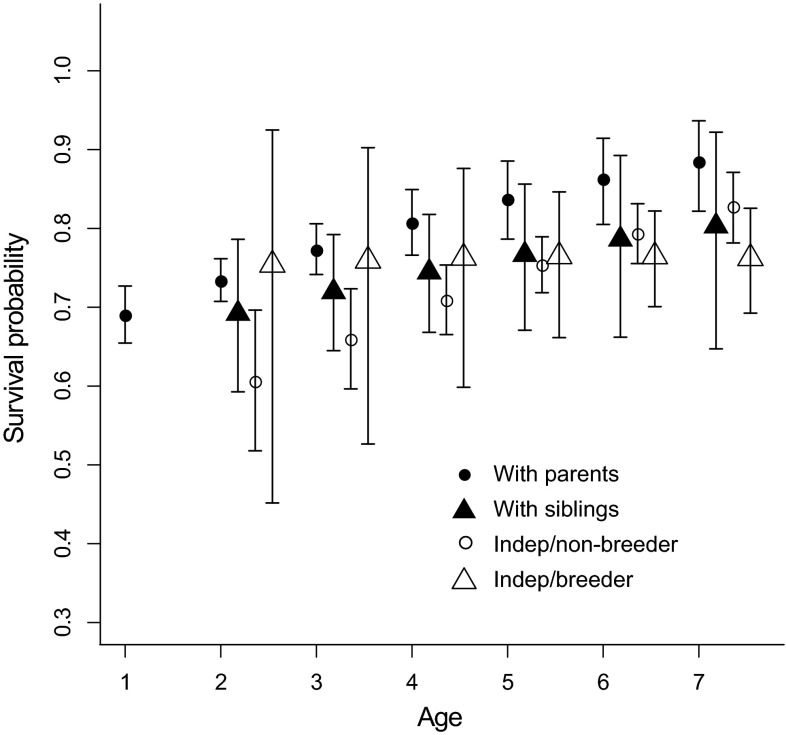
Mean posterior estimates (with 95 % credible intervals) of multistate survival in Greenland white-fronted geese with age (1–7+) modeled as a linear trend. States were with parents (*filled circles*), siblings (*filled triangles*), independent/nonbreeder (*open circles*), and independent/breeder (*open triangles*) at Wexford, Ireland in 1983–2003. Sibling, independent/nonbreeder, and independent/breeder states were estimated from age 2 because all birds began their capture history with at least one parent

**Fig. 3:**
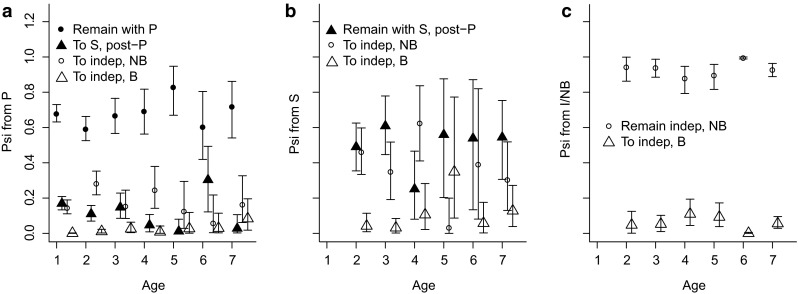
Mean posterior estimates of transition probabilities (psi; with 95 % credible intervals) for Greenland white-fronted geese with parents (*P*; **a**), siblings (*S*; **b**), and as independent/nonbreeders (*I/NB*; **c**). In each plot, transition probabilities are presented for all possible subsequent states; e.g., if starting with parents (**a**), possible states are remain with parents (*Remain with P*; *filled circles*), move to siblings (*To S*, *post-P*; *filled triangles*), move to independent/nonbreeder (*To I/NB*; *open circles*), or move to independent/breeder (*To I/B*; *open triangles*)

Survival of birds that were associated with parents was credibly greater than that of same-aged independent birds at all ages (i.e., 95 % credible intervals of birds with parents did not overlap means for birds considered independent/nonbreeders; Fig. 2). At ages 6 and 7, survival of birds with parents was also credibly greater than that of independent/breeder birds. In most cases, survival generally increased with age among all states, except for independent/breeder birds, whose age-specific survival was relatively stable across ages (Fig. 2). The probability of birds remaining with parents increased from age 2 (posterior mean = 0.60, 95 % CRI = 0.52–0.66) to age 5 (posterior mean = 0.83, 95 % CRI = 0.67–0.95), but decreased at age 6 (posterior mean = 0.61, 95 % CRI = 0.42–0.80), and increased slightly at age 7 (posterior mean = 0.72, 95 % CRI = 0.54–0.86; Fig. 3a). The probability of birds with siblings subsequently breeding successfully (i.e., skipping the independent/nonbreeder state) increased from age 3 (posterior mean = 0.03, 95 % CRI = 0.002–0.08) to age 5 (posterior mean = 0.35, 95 % CRI = 0.09–0.77), but decreased thereafter (Fig. 3b), and was credibly greater than the probability of independent birds subsequently breeding successfully at ages 5 (independent: posterior mean = 0.09, 95 % CRI = 0.04–0.17; *P* = 0.99), 6 (siblings: posterior mean 0.06, 95 % CRI = 0.002–0.18; independent: posterior mean 0.0004, 95 % CRI = 0.00001–0.01), and 7 (siblings: posterior mean 0.13, 95 % CRI = 0.04–0.27; independent: posterior mean 0.06, 95 % CRI = 0.03–0.10; see age-specific differences between open triangles in Fig. 3b, c). At all other ages, the probabilities of subsequently breeding successfully among individuals with siblings and those that were independent were generally similar, and 95 % CRI overlapped means. Resighting probability was the greatest among breeding individuals (posterior mean 0.94, 95 % CRI 0.89–0.97) and the least among independent, nonbreeding individuals (posterior mean 0.64, 95 % CRI 0.59–0.68).

### Cost–benefit matrix model

**Fig. 4:**
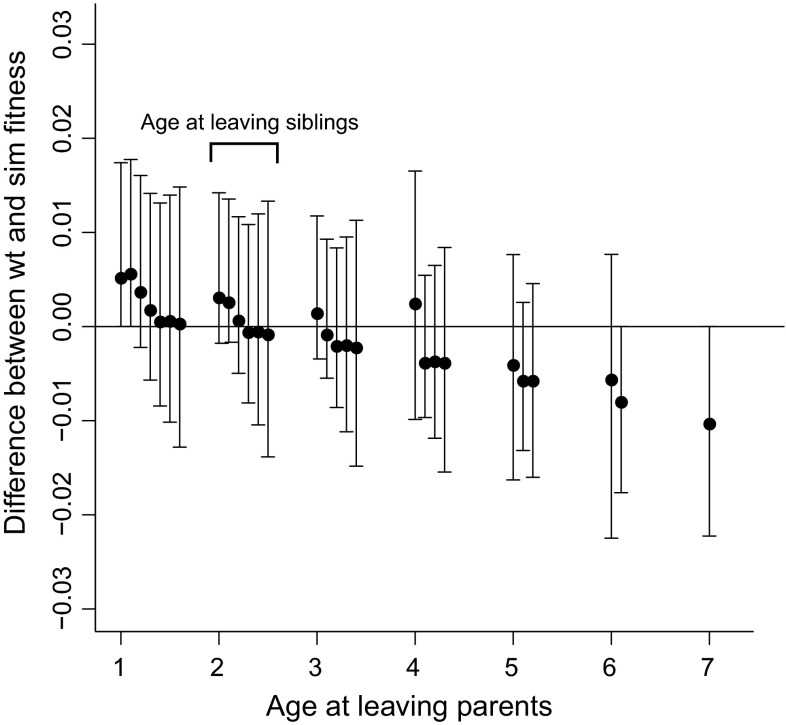
Age-specific (1–7+) fitness differences between wild-type (*wt*) and simulated (*sim*) distributions for birds leaving parents and siblings, calculated using 656 known-age Greenland white-fronted goose life histories. Ages at leaving siblings are represented by the multiple estimates at each age at leaving parents

The cost–benefit matrix model suggested that simulated fitness was credibly lower than wild-type fitness for birds that left parents and siblings at ages 1 (*P* = 0.02) and 2 (*P* = 0.007), and that simulated fitness was marginally lower than wild-type fitness for those that left parents and siblings at age 3 (*P* = 0.11) and those that left parents at age 1 and siblings at age 2 (*P* = 0.12; Fig. 4). These results suggest that offspring maintain familial association through age 3. At middle ages (i.e., ages 4–5), there were fewest differences between the simulated and wild-type fitness distributions, whilst at the oldest ages (i.e., ages 6–7+), simulated fitness (i.e., leaving parents or siblings) was generally greater than wild-type fitness among birds that remained with their parents for 5–6 years and siblings for 1–2 years (i.e., right-hand side of Fig. 4), which suggests that birds which remained with family in old age suffered lower fitness (although our sample sizes were small). A similar multistate model with age as a linear trend on survival but age-constant transition probabilities (Fig. S1 in the ESM) produced similar cost–benefit model results (Fig. S2 in the ESM).

## Discussion

This analysis shows that parent–offspring and sibling–sibling associations are beneficial for the first 3 years of life in Greenland white-fronted geese, whereby the fitness (the composite of survival and eventual breeding probability) of the birds that maintained such associations was credibly greater than the fitness of those that did not. Conversely, birds that maintained extended family associations (>3 years) gained no credible fitness benefit over individuals that left parents or siblings at the same age; further, our cost–benefit model provided weak evidence suggesting that fitness was actually lower among birds that remained with their parents or siblings than among simulated birds who were forced into independence at ages 6 and 7. These findings support the departure of individuals from the family unit at middle age (ages 4–5, where there were noncredible fitness differences between birds with parents or siblings and those that were independent) and old age. Although subsequent breeding probability was greatest for “older” individuals (those aged 5, as found in many other bird and some mammal species; Dobson 1982; Greenberg 1986; Wallace and Temple 1987; Sherry and Holmes 1989; Sedinger et al. 2001, 2008; Nichols et al. 2010) associating with siblings, these gains were offset by noncredible survival differences between birds with siblings and those that were independent, yielding lower overall fitness for birds aged 5 than those aged 3. Independence after just 2 or 3 years may be especially important for species characterized by very few breeders (such as Greenland white-fronted geese), as it allows younger individuals inherently more potential opportunities (than older birds who gain independence at older ages, all else being equal) to experience “optimal” breeding conditions. In the case of Greenland white-fronted geese, increased snowfall in Greenland has contributed to poorer breeding conditions and limited goose productivity there in recent years (Boyd and Fox 2008; Weegman 2014). These results are novel because previous work on parent–offspring associations has focused on the costs and benefits of shorter parental bonds (i.e., 1 or 2 years in duration; Cam et al. 2003; Nisbet et al. 1998; Tarwater and Brawn 2010) or the short-term costs and benefits of longer parental bonds (Inger et al. 2010), but very few studies have examined the lifetime fitness implications for offspring of long-term parental bonds in noncooperatively breeding birds where offspring maintain such family associations (with parents and siblings) into adulthood.

The cost–benefit model provides evidence for why most Greenland white-fronted geese exhibit relatively short family relationships (i.e., there is little fitness gained by associating with parents or siblings for longer than 2 or 3 years), but also why such variability exists in the duration of parental and sibling bonds; the decision between staying and leaving is marginally balanced after age 3 and does not favor one payoff over the other. The cost–benefit model provides one example from which we might suggest hypotheses about strategies in other animal populations as a consequence of differing life histories. For instance, if fitness was not a strong positive function of family bonds for the first few years of life, leaving parents and/or siblings earlier would likely be a preferable strategy. A similar model examining “staying” and “leaving” strategies for birds that exhibit shorter parent–offspring associations would confirm whether Greenland white-fronted geese exhibit a stronger “stay” payoff than others. One would assume this to be the case, as few bird species exhibit longer associations with parents.

In many animal populations, independence from the family unit is one of the most risky decisions in the life history of an individual. That individuals with parents enjoyed greater survival than independent/nonbreeders suggests a hidden cost of independence. Moreover, such costs accrued at all ages and were thus long-lasting for individuals independent at early ages; indeed, survival of independent/nonbreeders was lower than that of individuals with parents at all ages. Our comparison between simulated and wild-type fitness distributions showed no clear optimal fitness strategy after age 3, which implies a high degree of individual variation in this system, whereby individual condition likely influences the balance between the risk of dying before breeding and the increase in lifespan (and subsequent breeding probability) achieved by remaining with the family group; such heterogeneity could result in differential “optimal” fitness strategies.

The variation in individual “staying” and “leaving” strategies may be explained by whether parents or offspring determine association termination. For parents and offspring, maintaining the family bond is beneficial because larger family units are better able to defend resources and detect predators (Jarman 1974; Black and Owen 1989a; Gregoire and Ankney 1990; Tanner 2006). For parents, an additional benefit of larger group size is potentially greater success in future reproductive attempts (i.e., encouraging the “stay” strategy; Black and Owen 1989b). Yet, in geese, offspring do not form pair bonds or breed whilst associating with the family unit. Thus, if offspring determine optimal association duration, they may terminate bonds sooner to advance their direct fitness through reproductive attempts. In these cases, we would expect the “leave” strategy (i.e., simulated fitness) to be favored at earlier ages. In 173 cases (26 %) of 656 known-age geese, offspring were precipitated into independence, as parents were not seen again (i.e., having likely died). Hence, the majority (74 %) of parent–offspring associations were terminated based on choice by parents, offspring, or a combination of both. There are likely commonalities in perceived optimal association durations for parents and offspring, which may be driven by inclusive fitness benefits (Hamilton 1964). Indeed, “helping” among individuals increased survival and future reproductive success of recipients in other birds, for example in the Florida scrub jay (*Aphelocoma coerulescens*), pied kingfisher (*Ceryle rudis*), and splendid fairy-wren (*Malurus splendens*); Mumme et al. (1989). Although we did not specifically evaluate inclusive fitness in this system, prolonged associations may be favorable in this respect for parents and offspring in populations where very few individuals ever successfully breed, and most of those that do breed do so only once.

In this study, we have shown that maintaining family bonds for up to 3 years increases Greenland white-fronted goose offspring fitness, but that very few geese ever breed successfully; indeed, more than 90 % of known-age marked individuals were never observed on wintering areas with young. Thus, for most individuals, the reproductive benefits of family association and independence are not realized. Nonetheless, the survival benefits for individuals with parents compared to independent/nonbreeders provides a potential explanation for such extended family associations. Remarkably, two unpaired birds (of 14) that remained with their family into old age (7+ years) eventually bred, despite our cost–benefit model results suggesting lower fitness for such a strategy. Although not explicitly tested in this analysis, poor-quality birds that are unlikely to ever reproduce might also maintain family associations at older ages. For both highest- and lowest-quality individuals, remaining with the family unit may be an optimal life strategy for group size benefits (i.e., greater access to resources; Boyd 1953) and increased inclusive fitness if parents or siblings later reproduce. Although extended family associations are a feature of this population, they are relatively uncommon, and the survival benefits of such associations are not sufficient to yield clear fitness benefits. Therefore, extended associations only persist because parents and offspring mutually benefit from their persistence.

## Electronic supplementary material

Below is the link to the electronic supplementary material.

Supplementary material 1 (DOCX 93 kb)
