# Supplementary material for: Should I stay or should I go? Fitness costs and benefits of prolonged parent–offspring and sibling–sibling associations in an Arctic-nesting goose population
Source: Oecologia. 2016 Mar 19;181:809–17. doi: 10.1007/s00442-016-3595-4 (PMC4912589; doi:10.1007/s00442-016-3595-4)
Supplement: Supplementary file 1 — Supplementary material 1 (DOCX 93 kb) [file 442_2016_3595_MOESM1_ESM.docx]

**Electronic Supplementary Material**

**Should I stay or should I go? Fitness costs and benefits of prolonged parent-offspring and sibling-sibling associations in an Arctic-nesting goose population**

**Mitch D. Weegman, Stuart Bearhop, Geoff M. Hilton, Alyn J. Walsh, Kaitlin M. Weegman, David J. Hodgson, Anthony David Fox**

Corresponding author M.D. Weegman, e-mail: weegm009@umn.edu.

**Figure S1**

**Figure S2**

**Figure S3**

**Code for multistate model**

**Fig. S1** Mean posterior estimates of transition probabilities (with 95% credible intervals) for Greenland white-fronted geese from with parents (From P; green line), siblings (From S; red line) and independent/non-breeders (From I/NB; blue line) to with parents (P), siblings (S), independent/non-breeders (I/NB) and independent/breeders (I/B). For example, if starting with parents (green line), possible states are remain with parents (From P to P), move to siblings (From P to S), move to independent/non-breeder (From P to I/NB) or move to independent/breeder (From P to I/B)

**
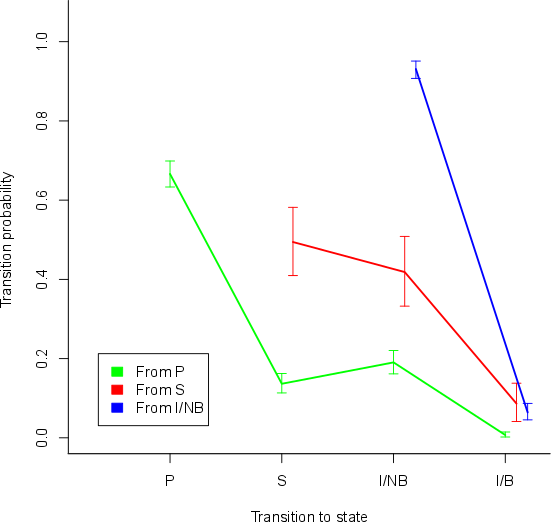
**

**Fig. S2** Age-specific (1-7+) fitness differences between wild type (wt) and simulated (sim) distributions for birds leaving parents and siblings, calculated using 656 known-age Greenland white-fronted goose life histories and estimated using a multistate model with age as a linear trend on survival and age-constant transition probabilities. Thus, all variation among ages leaving parents and siblings is due to variation in survival (and not transitions, which were age-constant). Ages of leaving siblings are represented by the multiple estimates at each age of leaving parents

**
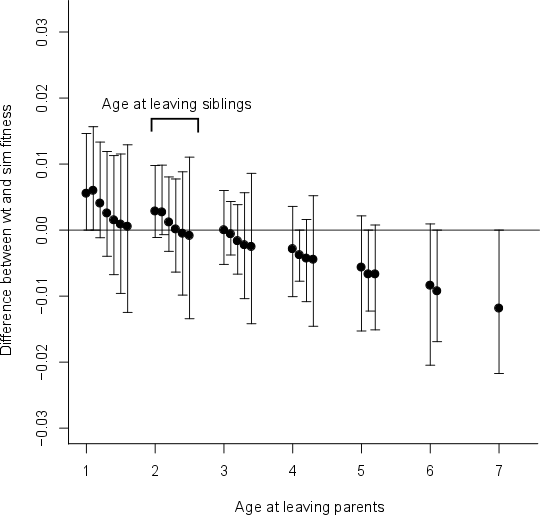
**

**Fig. S3** Longevity (years) of relationships exclusively with one sibling (i.e. after time with parents) in Greenland White-fronted Geese marked at Wexford, Ireland 1983-2003 (*n* subset above bars)

**Code for multistate model**

####################################

####################################

# Author: Mitch D. Weegman, adapting examples from Kery and Schaub 2012 and # Weegman et al. 2015

# Last modified: 07 January 2016

ms.history <- read.csv("brent multistate.csv", header = FALSE)

ms.history <- ms.history[,2:ncol(ms.history)]

# Create capture history matrix

CH <- as.matrix(ms.history)

# Compute vector with occasion of first capture

get.first <- function(x) min(which(x!=0))

f <- apply(CH, 1, get.first)

# Build age class matrix

a <- matrix(NA, ncol=dim(CH)[2]-1,nrow=dim(CH)[1])

for (i in 1:dim(CH)[1]){

a[i,f[i]:(dim(CH)[2]-1)]<-seq(1:(dim(CH)[2]-f[i]))

} #i

# Cap to age 7 across matrix

a <- ifelse(a > 7,7,a)

max.age<-7

CH <- ifelse(CH > 4,4,CH)

# Recode CH matrix

rCH <- CH

rCH[rCH==0] <- 5

library(R2WinBUGS)

bugs.dir<-"C:/WinBUGS14"

# Specify model in BUGS language

sink("goose-multinomlogit.bug")

cat("

model {

# -------------------------------------------------

# States:

# A: with parents

# B: with siblings

# C: independent/non-breeder

# D: independent/breeder

# Priors and constraints

# Survival and recapture: uniform

for (age in 1:max.age) {

phiA[age] <- 1/(1+1/exp(b0A+b1A*age))

phiB[age] <- 1/(1+1/exp(b0B+b1B*age))

phiC[age] <- 1/(1+1/exp(b0C+b1C*age))

phiD[age] <- 1/(1+1/exp(b0D+b1D*age))

}

pA ~ dunif(0, 1)

pB ~ dunif(0, 1)

pC ~ dunif(0, 1)

pD ~ dunif(0, 1)

b0A~dnorm(0,0.001)

b0B~dnorm(0,0.001)

b0C~dnorm(0,0.001)

b0D~dnorm(0,0.001)

b1A~dnorm(0,0.001)

b1B~dnorm(0,0.001)

b1C~dnorm(0,0.001)

b1D~dnorm(0,0.001)

# Transitions: multinomial logit

# Normal priors on logit of all but one transition probas

for (i in 1:3){

for (age in 1:max.age) {

lpsiA[age,i] ~ dnorm(0, 0.001)

lpsiB[age,i] ~ dnorm(0, 0.001)

lpsiC[age,i] ~ dnorm(0, 0.001)

lpsiD[age,i] ~ dnorm(0, 0.001)

}

}

# Constrain the transitions such that their sum is < 1

for (i in 1:3){

for (age in 1:max.age) {

psiA[age,i] <- exp(lpsiA[age,i]) / (1 + exp(lpsiA[age,1]) + exp(lpsiA[age,2]) + exp(lpsiA[age,3]))

psiB[age,i] <- exp(lpsiB[age,i]) / (1 + exp(lpsiB[age,1]) + exp(lpsiB[age,2]) + exp(lpsiB[age,3]))

psiC[age,i] <- exp(lpsiC[age,i]) / (1 + exp(lpsiC[age,1]) + exp(lpsiC[age,2]) + exp(lpsiC[age,3]))

psiD[age,i] <- exp(lpsiD[age,i]) / (1 + exp(lpsiD[age,1]) + exp(lpsiD[age,2]) + exp(lpsiD[age,3]))

}

}

# Calculate the last transition probability

for (age in 1:max.age) {

psiA[age,4] <- 1-psiA[age,1]-psiA[age,2]-psiA[age,3]

psiB[age,4] <- 1-psiB[age,1]-psiB[age,2]-psiB[age,3]

psiC[age,4] <- 1-psiC[age,1]-psiC[age,2]-psiC[age,3]

psiD[age,4] <- 1-psiD[age,1]-psiD[age,2]-psiD[age,3]

}

# Define state-transition and observation matrices

# Although all state transitions were included, impossible transitions (e.g., from independent back to with parents) yielded uninformative parameter estimates (95% credible intervals spanning from 0 to 1).

# Thus, such transitions were not included in the cost-benefit analysis.

for (i in 1:nind){

# Define probabilities of state S(t+1) given S(t)

for (t in f[i]:(n.occasions-1)){

ps[1,i,t,1] <- phiA[a[i,t]] * psiA[a[i,t],1]

ps[1,i,t,2] <- phiA[a[i,t]] * psiA[a[i,t],2]

ps[1,i,t,3] <- phiA[a[i,t]] * psiA[a[i,t],3]

ps[1,i,t,4] <- phiA[a[i,t]] * psiA[a[i,t],4]

ps[1,i,t,5] <- 1-phiA[a[i,t]]

ps[2,i,t,1] <- phiB[a[i,t]] * psiB[a[i,t],1]

ps[2,i,t,2] <- phiB[a[i,t]] * psiB[a[i,t],2]

ps[2,i,t,3] <- phiB[a[i,t]] * psiB[a[i,t],3]

ps[2,i,t,4] <- phiB[a[i,t]] * psiB[a[i,t],4]

ps[2,i,t,5] <- 1-phiB[a[i,t]]

ps[3,i,t,1] <- phiC[a[i,t]] * psiC[a[i,t],1]

ps[3,i,t,2] <- phiC[a[i,t]] * psiC[a[i,t],2]

ps[3,i,t,3] <- phiC[a[i,t]] * psiC[a[i,t],3]

ps[3,i,t,4] <- phiC[a[i,t]] * psiC[a[i,t],4]

ps[3,i,t,5] <- 1-phiC[a[i,t]]

ps[4,i,t,1] <- phiD[a[i,t]] * psiD[a[i,t],1]

ps[4,i,t,2] <- phiD[a[i,t]] * psiD[a[i,t],2]

ps[4,i,t,3] <- phiD[a[i,t]] * psiD[a[i,t],3]

ps[4,i,t,4] <- phiD[a[i,t]] * psiD[a[i,t],4]

ps[4,i,t,5] <- 1-phiD[a[i,t]]

ps[5,i,t,1] <- 0

ps[5,i,t,2] <- 0

ps[5,i,t,3] <- 0

ps[5,i,t,4] <- 0

ps[5,i,t,5] <- 1

# Define probabilities of O(t) given S(t)

po[1,i,t,1] <- pA

po[1,i,t,2] <- 0

po[1,i,t,3] <- 0

po[1,i,t,4] <- 0

po[1,i,t,5] <- 1-pA

po[2,i,t,1] <- 0

po[2,i,t,2] <- pB

po[2,i,t,3] <- 0

po[2,i,t,4] <- 0

po[2,i,t,5] <- 1-pB

po[3,i,t,1] <- 0

po[3,i,t,2] <- 0

po[3,i,t,3] <- pC

po[3,i,t,4] <- 0

po[3,i,t,5] <- 1-pC

po[4,i,t,1] <- 0

po[4,i,t,2] <- 0

po[4,i,t,3] <- 0

po[4,i,t,4] <- pD

po[4,i,t,5] <- 1-pD

po[5,i,t,1] <- 0

po[5,i,t,2] <- 0

po[5,i,t,3] <- 0

po[5,i,t,4] <- 0

po[5,i,t,5] <- 1

} #t

} #i

# Likelihood

for (i in 1:nind){

# Define latent state at first capture

z[i,f[i]] <- y[i,f[i]]

for (t in (f[i]+1):n.occasions){

# State process: draw S(t) given S(t-1)

z[i,t] ~ dcat(ps[z[i,t-1], i, t-1,])

# Observation process: draw O(t) given S(t)

y[i,t] ~ dcat(po[z[i,t], i, t-1,])

} #t

} #i

}

",fill = TRUE)

sink()

# Function to create known latent states z

known.state.ms <- function(ms, notseen){

# notseen: label for ‘not seen’

state <- ms

state[state==notseen] <- NA

for (i in 1:dim(ms)[1]){

m <- min(which(!is.na(state[i,])))

state[i,m] <- NA

}

return(state)

}

# Function to create initial values for unknown z

ms.init.z <- function(ch, f){

for (i in 1:dim(ch)[1]){ch[i,1:f[i]] <- NA}

states <- max(ch, na.rm = TRUE)

known.states <- 1:(states-1)

v <- which(ch==states)

ch[-v] <- NA

ch[v] <- sample(known.states, length(v), replace = TRUE)

return(ch)

}

# Bundle data

bugs.data <- list(y = rCH, f = f, n.occasions = dim(rCH)[2], nind = dim(rCH)[1], z = known.state.ms(rCH, 5), max.age = max.age, a = a)

# Initial values

inits <- function(){list(b0A = rnorm(1, 0, 1), b0B = rnorm(1, 0, 1),b0C = rnorm(1, 0, 1),b0D = rnorm(1, 0, 1),b1A = rnorm(1, 0, 1),b1B = rnorm(1, 0, 1),b1C = rnorm(1, 0, 1),b1D = rnorm(1, 0, 1), lpsiA = array(rnorm(28),dim=c(max.age,3)), lpsiB = array(rnorm(28),dim=c(max.age,3)), lpsiC = array(rnorm(28),dim=c(max.age,3)), lpsiD = array(rnorm(28),dim=c(max.age,3)), pA = runif(1, 0, 1) , pB = runif(1, 0, 1) , pC = runif(1, 0, 1), pD = runif(1, 0, 1), z = ms.init.z(rCH, f))}

# Parameters monitored

parameters <- c("b0A","b0B","b0C","b0D","b1A","b1B","b1C","b1D","phiA", "phiB", "phiC", "phiD", "psiA", "psiB", "psiC", "psiD", "pA", "pB", "pC", "pD")

# MCMC settings

ni <- 450000

nt <- 10

nb <- 90000

nc <- 3

# Call WinBUGS from R

goose.ms <- bugs(bugs.data, inits, parameters, "goose-multinomlogit.bug", n.chains = nc, n.thin = nt, n.iter = ni, n.burnin = nb, debug = TRUE, bugs.directory = bugs.dir, working.directory = getwd())

# Summarize posteriors

goose.summary <- print(goose.ms, digits = 3)

####################################

####################################
